# Case report: Functional analysis of the p.Arg507Trp variant of the *PIGT* gene supporting the moderate epilepsy phenotype of mutations in the C-terminal region

**DOI:** 10.3389/fneur.2023.1092887

**Published:** 2023-03-09

**Authors:** Ikhlas Ben Ayed, Olfa Jallouli, Yoshiko Murakami, Amal Souissi, Salma Mallouli, Amal Bouzid, Fatma Kamoun, Ines Elloumi, Fakher Frikha, Abdelaziz Tlili, Sarah Weckhuysen, Taroh Kinoshita, Chahnez Charfi Triki, Saber Masmoudi

**Affiliations:** ^1^Laboratory of Molecular and Cellular Screening Processes (LPCMC), Center of Biotechnology of Sfax, University of Sfax, Sfax, Tunisia; ^2^Medical Genetics Department, University Hedi Chaker Hospital of Sfax, Sfax, Tunisia; ^3^Child Neurology Department, University Hedi Chaker Hospital of Sfax, Sfax, Tunisia; ^4^Research Laboratory “Neuropédiatrie” LR19ES15, Sfax University, Sfax, Tunisia; ^5^Laboratory of Immunoglycobiology, Research Institute for Microbial Diseases, Osaka University, Suita, Japan; ^6^Department of Applied Biology, College of Sciences, University of Sharjah, Sharjah, United Arab Emirates; ^7^Human Genetics and Stem Cell Laboratory, Research Institute of Sciences and Engineering, University of Sharjah, Sharjah, United Arab Emirates; ^8^Department of Neurology, University Hospital, Antwerp, Belgium; ^9^Center for Infectious Disease Education and Research, Osaka University, Suita, Japan

**Keywords:** *PIGT* gene, global developmental delay, epilepsy, genotype phenotype correlation, GPI transamidase

## Abstract

Pathogenic germline variants in the *PIGT* gene are associated with the “multiple congenital anomalies–hypotonia-seizures syndrome 3” (MCAHS3) phenotype. So far, fifty patients have been reported, most of whom suffer from intractable epilepsy. Recently, a comprehensive analysis of a cohort of 26 patients with *PIGT* variants has broadened the phenotypical spectrum and indicated that both p.Asn527Ser and p.Val528Met are associated with a milder epilepsy phenotype and less severe outcomes. Since all reported patients are of Caucasian/Polish origin and most harbor the same variant (p.Val528Met), the ability to draw definitive conclusions regarding the genotype–phenotype correlation remains limited. We report a new case with a homozygous variant p.Arg507Trp in the *PIGT* gene, detected on clinical exome sequencing. The North African patient in question displays a predominantly neurological phenotype with global developmental delay, hypotonia, brain abnormalities, and well-controlled epileptic seizures. Homozygous and heterozygous variants in codon 507 have been reported to cause *PIGT* deficiency without biochemical confirmation. In this study, FACS analysis of knockout HEK293 cells that had been transfected with wild-type or mutant cDNA constructs demonstrated that the p.Arg507Trp variant leads to mildly reduced activity. Our result confirm the pathogenicity of this variant and strengthen recently reported evidence on the genotype–phenotype correlation of the *PIGT* variant.

## Introduction

It is estimated that 10–20% of all membrane proteins are post-translationally modified at their C-terminus by glycosylphosphatidylinositol (GPI), a complex glycophospholipid that anchors over 150 proteins to the cell surface. The transfer of the GPI anchor to proteins is catalyzed by the GPI transamidase (GPI-TA) complex (1, 2). Phosphatidylinositol glycan anchor biosynthesis class T (*PIGT*) is a highly conserved 578-amino-acid protein. *PIGT* interacts with other components of the GPI-TA complex (PIGK, PIGS, *PIGT*, PIGU, and PGAA1) and appears to be the most important protein in the formation of this complex and the stability of the other components (3, 4). Disorders caused by germline pathogenic variants in *PareT* are referred to as multiple congenital anomalies–hypotonia-seizures syndrome 3 (MCAHS3, OMIM#615398). This syndrome is characterized by congenital hypotonia, global developmental delay (GDD) or intellectual disability (ID), and infantile-onset epilepsy with various types of epileptic EEG abnormalities (focal sharp-slow wave, focal sharp spike/polyspikes wave, and generalized polyspikes-wave complexes) (5, 6). Other congenital anomalies involving dysmorphic facial features, cerebral and cerebellar atrophy, and defects in the skeletal, ophthalmological, cardiac, and genitourinary systems are also reported (7–9).

Up to now, 50 patients with *PIGT* deficiency, most of them Caucasian, have been diagnosed with 17 pathogenic variants, including 10 missense, one non-sense, four frameshifts, and two splice sites. The largest extant genotype–phenotype study, reviewed by Bayat et al. reports on 26 patients with the p.Val528Met variant in either homozygous or compound heterozygous state and on a single patient who had the p.Asn527Ser variant (10, 11). These patients presented with moderate to severe GDD and later onset of epilepsy, and generally became seizure-free on monotherapy. Since all reported patients thus far have been of Caucasian/Polish origin and most harbor the same variant (p.Val528Met), the ability to draw definitive conclusions regarding the genotype–phenotype correlation remains limited. Here, we provide additional findings supporting this correlation by reporting a new case from North Africa, of a patient carrying the *PIGT* p.Arg507Trp variant located in the C-terminal region and associated with GDD and partially tractable epilepsy. Functional studies using *PIGT* knockout HEK293 cells showed mildly decreased activity of the GPI-TA complex, supporting the pathogenicity of the variant and providing further evidence of an association between the localization of variants in the C-terminal region and the moderate epilepsy phenotype (seizure-free or partially seizure-free on treatment).

## Patient and methods

We reviewed the patient's personal history regarding progress of the pregnancy and childbirth, psychomotor milestone achievement, and neurological examinations. We also reviewed the history of epileptic seizures (type, age of onset, response to antiseizure medication), electroencephalographic data (background, epileptiform discharges, epileptic seizure if recorded), and brain imaging data, as well as the etiological assessment.

For the genetic study, DNA was isolated from peripheral blood and clinical exome sequencing was performed using the TruSight™ One Sequencing Panel. Libraries were prepared and data analysis was performed as previously described (12). The generated VCF file was annotated using the VarAft application (13). A virtual gene panel was applied using the Genetic Epilepsy Syndromes panel from Genomics England PanelApp (https://panelapp.genomicsengland.co.uk/panels/). The variant-filtering process consisted of the following four steps: (i) selection of variants localized in exons or bordering introns (±12 bp); (ii) exclusion of variants with a frequency above 1% in general populations; (iii) selection of variants predicted as pathogenic by CADD; and (iv) selection of variants predicted as pathogenic by the UMD-Predictor tool. *In-silico* pathogenicity was also evaluated using the VarCards tool (http://varcards.biols.ac.cn/). The preferentially selected variants were validated and familial co-segregation was analyzed *via* Sanger sequencing.

The study protocol was approved by the local medical ethics committee of South Tunisia (Accession number 28/2019). Written informed consent was obtained from the patient's parents.

*PIGT*-knockout HEK293 cells were generated and transfected, as described previously, with human wild-type or mutant *PIGT* cDNA cloned into pTA, a weak promoter (TA promoter-driven expression vector) that helps with the detection of mild partial loss of function (14). Two days later, restoration of GPI-AP (CD59, DAF, and CD16) expression was measured *via* flow cytometry. Cells were stained with PE-conjugated anti-human CD16 antibody (3G8, Biolegend) along with mouse anti-human CD59 (5H8) and mouse anti-human DAF (IA10) antibodies, followed by PE-conjugated second antibody. Levels of expressed wild-type and p.Arg507Trp mutant HA-tagged *PIGT* in pME-vector transfected cells were analyzed by western blotting using an anti-HA antibody (C29F4, Cell Signaling Tec, Danvers, MA, USA). Levels of protein expression were normalized to luciferase activity for transfection efficiencies and to expression of GAPDH for loading controls.

## Results

### Clinical data

**Figure 1:**
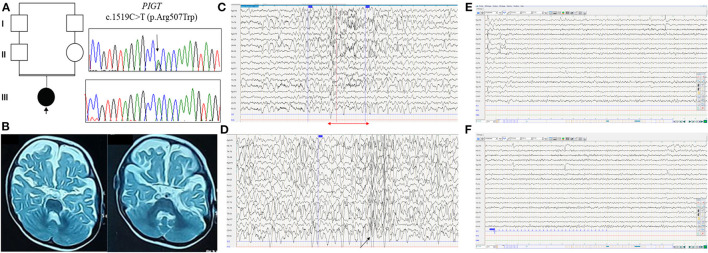
Clinical and molecular findings in the Libyan family with *PIGT* variant c.1519C>T; p.Arg507Trp. **(A)** Pedigree of the reported family with electropherograms of the identified homozygous *PIGT* variant (III.1) compared with heterozygous carrier sequences (II.1 and II.2). **(B)** Brain MRI T2-weighted axial images reveal severe atrophy of the cerebral hemispheres with slight cerebellar hypoplasia. **(C)** EEG recording, obtained when the patient was 2 years old, with the following parameters: 20 electrodes, 7 μv/mm, 0.03 s, 15, 50 Hz. The EEG shows slow spike-wave discharge followed by diffuse fast rhythms (red arrow). **(D)** Sleep EEG shows moderately organized EEG with the presence of rare spindles and diffuse spike-wave discharges (black arrow). **(E, F)** EEG showing normal background rhythm without photosensitivity.

The proband, a Libyan girl born in 2017, was the first child of healthy consanguineous parents (Figure 1A). She was born at full term, and pregnancy and delivery were uneventful with normal menstruation. Global hypotonia and horizontal nystagmus were noted from the neonatal period. At the age of 4 months, the patient presented with multiple (two to three) daily seizures with asymmetric adduction movement of the 4 limbs and head deviation. The ictal sleep EEG showed asymmetric tonic spasm (Figure 1B). The interictal sleep EEG showed organized EEG with the presence of rare spindles and diffuse spike-wave discharges (Figure 1C). The patient presented with a global psychomotor delay with hypotonia, a severe speech delay, and walking impairment. Brain MRI, performed at age 14 months, revealed severe atrophy of the cerebral hemispheres with slight cerebellar hypoplasia and enlargement of lateral ventricles (Figure 1D). Metabolic screening was normal. She was treated with corticosteroid for 2 weeks (hydrocortisone 15 mg/kg/day) without improvement; hence, she was switched to vigabatrin (100 mg/kg/day) in association with valproate (30 mg/kg/day). Her overall antiepileptic drug response was good, but she still experienced seizures once every 2 weeks.

Based on clinical data acquired during the most recent follow-up at the age of 2 years 6 months, our patient showed severe motor and cognitive retardation: she cannot hold her head up or sit, she has no eye tracking, with wandering movements of the eyeballs, expressive language is completely absent, and she has axial hypotonia with spastic tetraparesis. She also has dysmorphic features, including a broad forehead, acquired microcephaly, deep-set eyes, epicanthus, and high-arched palate. At the most recent follow-up, the patient had normal sleep EEG with normal background rhythm without photosensitivity (Figures 1E, F).

### Molecular results

A total of 9,542 common single-nucleotide variants (SNVs) and indels were detected. The filtering process, as previously described, resulted in identification of 13 homozygous and 118 heterozygous variants. Based on their predicted effect on protein function and the likelihood of the autosomal recessive mode of inheritance, we evaluated the resulting variants, under the assumption that the disease gene would be more likely to cause an epileptic and/or a DD phenotype. Initially, only homozygous variants were considered and the *PIGT* variant (NM_015937.6):c.1519C>T (p.Arg507Trp) was selected as the candidate variant for the patient's phenotype. For the purpose of further investigation, we reevaluated the filtering process considering an autosomal dominant mode of inheritance; however, no relevant variants were detected. The *PIGT* variant was verified *via* Sanger sequencing, and both asymptomatic parents were found to be heterozygous (Figure 1A). The identified missense variant (rs146484791) was reported with a frequency of 0.0023% in the gnomAD database and has not been previously reported in the homozygous state. *In-silico* pathogenicity prediction using the VarCards tool showed a deleterious effect on the *PIGT* protein (damaging score 0.83), with a damaging CADD score of 35. An alternative variant, p.Arg507Gln, was found to have been reported in the heterozygous state with p.Val528Met in two patients with *PIGT* deficiency (10, 11).

### Functional analysis of the *PIGT* variant

**Figure 2:**
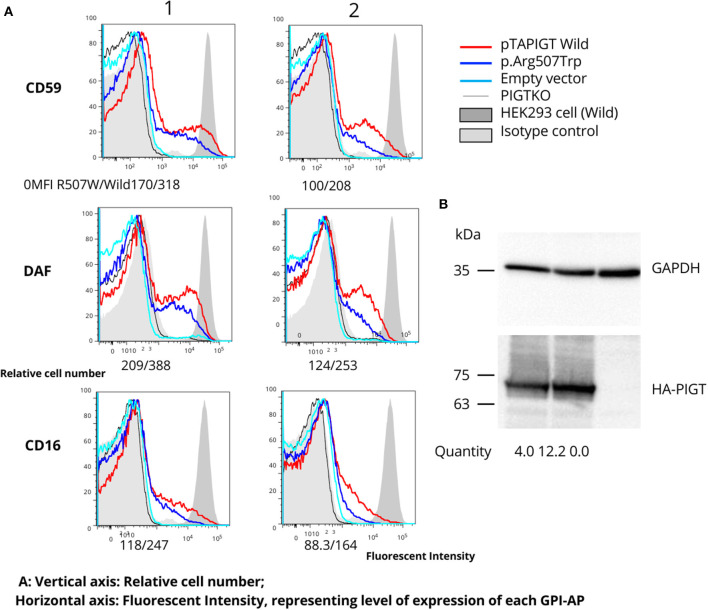
Follow-up functional studies on the p.Arg507Trp variant. **(A)** *PIGT* knockout HEK293 cells were transfected with variable amounts of p.Arg507Trp-variant *PIGT* or wild-type *PIGT* expressing plasmids driven by the weak promoter (pTA; 1: 4 ug; 2: 0.8 ug). Restoration of expression of GPI-APs (CD59, DAF, and CD16) was measured using flow cytometry. The p.Arg507Trp variant could not restore GPI-APs at a similar level to wild-type *PIGT*. The vertical axis represents the relative cell number; the horizontal axis represents fluorescent intensity, reflecting the level of expression of each GPI-AP. **(B)** Levels of expressed wild-type and p.Arg507Trp mutant HA-tagged *PIGT* in pME vector-transfected cells were analyzed by western blotting using an anti-HA antibody. After normalization to luciferase activity and GAPDH, expression of the mutant protein appeared to be similar to that of the wild-type protein.

Rescue experiments using variant cDNA driven by the weak promoter pTA, performed on *PIGT*-knockout HEK293 cells, indicated that the p.Arg507Trp variant results in a mild reduction in the amounts of CD59, CD16, and DAF anchored to the cell membrane (Figure 2A). Western blot analysis showed that expression of the mutant protein was not decreased but was even higher than expression of the wild-type protein (Figure 2B).

Taking these findings together, we classified the Arg507Trp as a likely pathogenic variant in accordance with the ACMG recommendations: PM2 (variant not found in gnomAD genomes with good coverage of gnomAD genomes = 29.9; pathogenic, supporting), PM5 (alternative pathogenic variant Arg507Gln had been already reported (10, 11); pathogenic, moderate), and PS3 (well-established *in vitro* functional studies supportive of a damaging effect of the gene product; pathogenic, strong).

## Discussion

Clinical exome sequencing is now used routinely as a tool to diagnose many different inborn errors of metabolism (IEMs) in patients with developmental and epileptic encephalopathy (DEE), such as congenital disorders of glycosylation. However, despite the tremendous progress that has been made in the development of both sequencing technology and bioinformatics in recent years, it should be noted that other diagnostic tests are still necessary to confirm or reject a diagnosis with certainty in cases where this approach results in the identification of genetic variants of uncertain clinical significance (VOUS). The variant p.Arg507Trp in the *PIGT* gene, identified in the homozygous state in this study, has previously been reported in a compound heterozygous state with Val528Met in two Russian patients (11). In addition, an alternative variant in the same location (Arg507Gln) has been reported in a Polish patient (10). According to ACMG classification, this variant is still classified as a VOUS, since it has not been biologically confirmed. The functional experiments performed here using mutant HEK293 cells provide evidence to support the pathogenicity of this variant; this evidence is sufficient to reclassify this variant as likely pathogenic. On the other hand, the milder decreased activity is consistent with the moderate phenotype of our patient. Here we should highlight that one of the limitations of our study is the brief period of follow-up of our patient, who is only at the age of 2 years at the time of writing. However, we were able to identify our patient's phenotype specifically as a moderate epileptic one in comparison to the majority of *PIGT*-deficiency patients, who often suffer from severe drug-resistant epilepsy with convulsive status epilepticus, sometimes with neonatal–infantile onset. Together with other recent studies (10, 11), our study also serves to confirm the genotype–phenotype correlation for *PIGT* that was first described in 2019. Bayat et al. report on 24 patients harboring the p.Val528Met variant in either homozygous or compound heterozygous state and one patient with the p.Asn527Ser variant, all of whom presented with a less severe epilepsy phenotype with considerably later onset of epilepsy; there was also no premature mortality in any of these patients (11). In Table 1, we present a comparison of the clinical findings for our patient with those of previously reported patients with the p.Arg507Trp variant. All patients presenting with the p.Arg507Trp variant in either homozygous or compound heterozygous state, including our patient, have been found to exhibit GDD and became free or partially free of epileptic seizures following treatment with CBZ/VPA. Additionally, a comparison of the phenotypes of patients harboring both p.Asn527Ser and p.Val528Met variants with that of our patient revealed a similar spectrum of symptoms regarding the onset of seizures (between 4 months and 5.5 years), seizure types (focal to bilateral tonic–clonic seizures as the predominant type), degree of cognitive and developmental outcome (moderate to severe), and response to antiepileptic drugs. Therefore, we conclude that missense variants p.Asn527Ser, p.Val528Met, and p.Arg507Trp, located in the C-terminal region, are associated with an epileptic phenotype with comparatively better outcome.

**Table 1:** Brief clinical overview of all individuals with *PIGT* deficiency associated with moderate seizure phenotype of variants within the C-terminal region (*PIGT*: NM_015937.6) for whom clinical information has been published.

	**Group 1: Individuals with at least one variant at p.Arg507**				**Group 2: Individuals (*n* = 25) with either the p.Val528Met or the p.Asn527Ser variant**
	**p.Arg507Trp; p.Val528Met**		**p.Arg507Gln; p.Val528Met**	**p.Arg507Trp; p.Arg507Trp**	**Patients with compound missense/truncating (*****n*** = **10), biallelic missense (*****n*** = **15), or biallelic truncating (*****n*** = **0) variants**
References	[(11); Family 7]		(10)	Current study	(11)
Number of patients	2		1	1	25
Geographical/ethnic origin	Russian		Polish	Libyan (North African)	8 Caucasian; 2 Caucasian/African; 1 Asian; 11 Polish; 3 Russian
	Patient 7	Patient 8	P3	Reported patient	
Age at inclusion	8 years	22 years	Born 2017	4 years	2–28 years
Febrile seizures	No	No	Yes	No	No: 7/19; Yes: 12/19
Epilepsy diagnosis	Yes	Yes	Yes	Yes	Yes: 17/25; No: 8/25
Status epilepticus	No	No	Unknown	No	No: 11; Not relevant: 2; Yes: 1; Unknown: 11
Age of seizure onset	5 months	18 months	6 months	4 months	Range: 5 months to 5.5 years
Seizure types during disease course	Myoclonic atonic epilepsy; eyelid myoclonia	Myoclonic atonic epilepsy	Focal hypomotor seizures with impaired awareness, focal to bilateral tonic–clonic seizure	Focal to bilateral tonic–clonic seizure	Atypical absences: 2; Fever-induced tonic–clonic/focal seizures: 4; Focal hypomotor, seizures with impaired awareness, FBTCS: 6; Bilateral tonic–clonic seizures: 2; Focal to bilateral tonic–clonic seizure: 4; MAE: 2; Unknown:1; Not relevant: 2
Overall antiepileptic drug response	Very good	Very good	Very good	Very good	Good (24/25, 96% seizure-free)
Degree of developmental delay	Severe	Severe	Moderate	Severe	Moderate (5/25), moderate–severe (6/25), severe (4/25), unable to ascertain (10/25)
Congenital hypotonia	Yes	Yes	Yes	Yes	24/25
Brain MRI results	10 months: Delayed myelination and enlargement of the subarachnoid spaces	15 years: Cerebellar atrophy and thinning of the corpus callosum	12 months: Decreased white matter volume, enlargement of pericerebral spaces	14 months: Atrophy of the cerebral hemispheres with slight cerebellar hypoplasia and enlargement of lateral ventricles	Delayed myelination: 7; Cerebellar atrophy: 11; Cortical atrophy: 4; Not performed: 7.

FBTCS, Focal to bilateral tonic–clonic seizure; MAE, Myoclonic atonic epilepsy.

**Figure 3:**
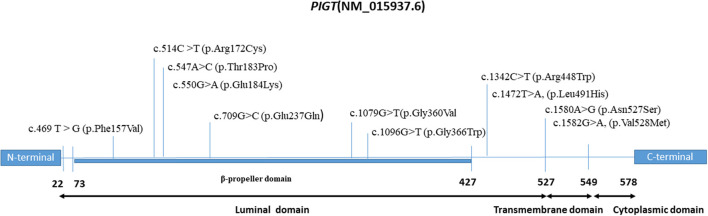
Domain map of the *PIGT* protein depicting the locations of milder and severe pathogenic variants.

The existing literature was also searched to identify all missense variants described as occurring with a severe phenotype, including severe epilepsy with frequently recurrent episodes of convulsive status epilepticus, abnormal interictal EEG, and severe drug-resistant epilepsy. In total, 10 missense variants (either in homozygous or in compound heterozygous state) were collected; these have been observed in 23 patients belonging to 14 families of various ethnicities (Table 2). Interestingly, we note that all of these variants occur in the N-terminal region of the *PIGT* protein (Figure 3). Hence, we suggest that the phenotypic variability of *PIGT* deficiency could be related to the position of the affected residue and its effect on the function or structure of the *PIGT* protein, as well as the interaction with components of the GPI-TA complex. It has been shown that *PIGT* is covalently linked to the majority of PIGK *via* a functionally important disulfide bond between *PIGT*-Cys182 and PIGK-Cys92 in the GPI-TA complex. In addition, *PIGT* and PIGS are linked to one other, and *PIGT* has an additional function in stabilizing GPI-TA by linking PIGS to GAA1 and PIGK (3, 4). In the case of the homozygous p.Thr183Pro and p.Glu184Lys variants, substitutions occur in the residue directly adjacent to Cys182, which is responsible for the *PIGT*–PIGK disulfide bond. In the p.Thr183Pro variant, the threonine is substituted by a proline, which is an inflexible residue common in very tight protein junctions (5). In the p.Glu184Lys variant, glutamate (negatively charged) is substituted by a positively charged lysine. These two substitutions could affect the biochemical environment around Cys182, possibly affecting the stability of the disulfide bond leading to loss of function in *PIGT* and the GPI-TA complex. Considering the available experimental data, a recent study has additionally suggested that the luminal part of *PIGT* (73-427 aa) consists of a β-propeller domain with a central hole that regulates the access of substrate protein C-termini to the active site of the cysteine protease PIGK (19). Therefore, missense variants located in the β-propeller domain could affect the interaction of *PIGT* with the active site of PIGK, leading to the observed severe phenotype.

**Table 2:** Published clinical findings in individuals harboring a severe phenotype of *PIGT* deficiency associated with missense variants.

**Missense variants**	**c.709G**>**C (p.Glu237Gln)**		**c.547A>C (p.Thr183Pro)**	**c.550G**>**A (p.Glu184Lys)**		**c.1079G**>**T (p.Gly360Val)**			**c.1342C>T (p.Arg448Trp)/ c.250G>T (p.Glu84Ter)**	**c.1342C>T (p.Arg448Trp)/ c.918dupC (p.Val307 ArgfsTer13)**	**c.250G>T/ (p.Glu84Ter) c.1096G>T/ (p.Gly366 Trp)**	**c.1472T>A (p.Leu491His)/ 1484+2T>A**	**c.469T>G (p.Phe157Val)/ c.1120A>G (p.Asn374Asp)**	**c.514C>T (p.Arg172Cys)/ c.98delA (p.Glu33AspfsTer12)**
No. families	2		1	2		3			1	1	1	1	1	1
References	(14)	(6)	(5)	(15)	(6)	(16)	(6)	(6)	(9)	(7)	(17)	(6)	(18)	(18)
Geographical/ ethnic origins (no. patients)	Afghani (1)	Bangladeshi (2)	Turkish (4)	Chinese (1)	Pakistani (1)	African (2)	Somalian (2)	- (2)	Japanese	Caucasian/ African American (2)	Japanese (1)	Danish (2)	Chinese (1)	Chinese (1)
Age of onset of epilepsy	Neonatal onset	1st day; 2 weeks	12–18 months	1 month	5 months	12 months	11–14 months	11–14 months	4 months	5 months	2 months	1st day	3 months	4 months
Seizure types during disease course	Generalized tonic–clonic	Fever-induced myoclonic and subtle focal; generalized tonic seizures	Myoclonic (2/4); generalized tonic–clonic (1/4)	Myoclonic and febrile seizure	Generalized tonic and myoclonic; focal myoclonic seizures; febrile seizures	Myoclonic, tonic, and tonic–clonic seizures that occasionally generalize	Generalized tonic–clonic and atonic seizures; myoclonic and focal seizures	Myoclonic and subtle focal seizures; febrile seizures	Myoclonic tonic with apnea that can generalize	Myoclonic, tonic, and tonic–clonic seizures that occasionally generalize	Myoclonic, tonic with apnea that can generalize	Myoclonic and febrile seizure; tonic and myoclonic with apnea; subtle focal seizures	Febrile/ afebrile seizures	Clonic afebrile seizures
Overall seizure outcome	Intractable	Intractable	Intractable	Intractable	Intractable	Intractable	Intractable (seizure-free on ketogenic diet for 1 patient)	Intractable	Intractable	Intractable	Intractable	Intractable	Intractable	Intractable
Degree of DD	Severe	Severe	Severe	Severe	Severe	Severe	Severe	Severe	Severe	Severe	Severe	Severe	Severe	Severe
Premature mortality	-	1 patient deceased (6 months)	-	-	-	-	-	1 patient deceased (26 months)	-	-	-	1 patient deceased (26 months)	-	-

-, data not reported.

Concerning variants occurring in the C-terminal region on the cytoplasmic side (p.Val528Met, p.Asn527Ser, and p.Arg507Trp), we suggest that these variants would not affect the *PIGT*-GPI-APs interaction, instead representing residual activity, which might explain the moderate phenotype. This hypothesis can be supported by the results of our rescue experiments using variant cDNA driven by the weak promoter pTA on *PIGT*-knockout HEK293 cells. Specifically, p.Arg507Trp variant did not restore the GPI-APs to a similar level as observed with wild-type *PIGT*, suggesting decreased activity of the variant. In addition, the same effect has also been demonstrated for the p.Val528Met variant, although this effect was only observed when the pTK promoter was used (14).

Our study details a new case of the p.Arg507Trp variant in the C-terminal region, associated with a moderate epileptic phenotype. This supports the previously reported genotype–phenotype correlation of *PIGT* deficiency (10, 11), which will be useful for future genetic counseling.

## Data availability statement

The datasets presented in this article are not readily available because of ethical/privacy restrictions. Requests to access the datasets should be directed to the corresponding author.

## Ethics statement

The study protocol was approved by the Local Medical Ethics Committee of South of Tunisia (Accession number 28/2019). Written informed consent to participate in this study was provided by the participants' legal guardian/next of kin. Written informed consent was obtained from the individual(s), and minor(s)' legal guardian/next of kin, for the publication of any potentially identifiable images or data included in this article.

## Author contributions

IB conceived the study, wrote the manuscript, and analyzed and interpreted the NGS data. OJ, SMal, and FK analyzed and interpreted all clinical data. AT, AS, and AB conducted the clinical exome sequencing. YM performed the functional study. IE performed a segregation analysis. FF and AS assisted in writing the manuscript. SMas, SW, TK, and CT revised and edited the manuscript. All authors contributed to the article and approved the submitted version.
